# A self-marker-like protein governs hemocyte allorecognition in *Halocynthia roretzi*

**DOI:** 10.1186/s40851-019-0149-8

**Published:** 2019-12-16

**Authors:** Masaki Ema, Taizo Okada, Miki Takahashi, Masato Uchiyama, Hideo Kubo, Hideaki Moriyama, Hitoshi Miyakawa, Midori Matsumoto

**Affiliations:** 1 Department of Biological Sciences and Informatics, Keio University, 3-14-1, Hiyoshi, Kouhoku-ku, Yokohama, 223-8522 Japan; 2 Tokyo Metropolitan Institute of Medical Science, 2-1-6, Kami-Kitazawa, Setagaya-ku, Tokyo, 156-8506 Japan; 3 School of Biological Sciences, University of Nebraska-Lincoln, Lincoln, NE USA; 4 Laboratory of Environmental Physiology, Center for Bioscience Research and Education, Utsunomiya University, 350 Mine-machi, Utsunomiya, Tochigi, 321-8505 Japan

**Keywords:** Allorecognition, Ascidian, *Halocynthia roretzi*, Contact reaction, Hemocyte

## Abstract

**Background:**

Self-incompatibility, fusion/non-fusion reactions, and contact reactions (CRs) have all been identified as allorecognition phenomena in ascidians. CR is a reaction characteristic of the hemocytes of *Halocynthia roretzi*, whereby they release phenol oxidase (PO) upon contact with non-self hemocytes. Thus, these cells may represent a primitive form of the vertebrate immune system. In the present study, we focused on the CR of *H. roretzi* hemocytes and sought to identify self-marker proteins that distinguish between self and non-self cells.

**Results:**

We initially generated a CR-inducing monoclonal antibody against the complete hemocyte membrane-protein complement (mAb11B16B10). This antibody was identified based on the differential induction of PO activity in individual organisms. The level of PO activity induced by this antibody in individual ascidians was consistent with the observed CR-induced PO activity. mAb11B16B10 recognized a series of 12 spots corresponding to a 100-kDa protein, with differing isoelectric points (pIs). A comparison of the 2D electrophoresis gels of samples from CR-reactive/non-reactive individuals revealed that some spots in this series in hemocytes were common to the CR-non-inducible individuals, but not to CR-inducible individuals. We cloned the corresponding gene and named it *Halocynthia roretzi* self-marker-like protein-1 (HrSMLP1). This gene is similar to the glycoprotein DD3–3 found in *Dictyostelium,* and is conserved in invertebrates.

**Conclusion:**

We generated a CR-inducing monoclonal antibody (mAb11B16B10) that recognized a series of novel membrane proteins (HrSMLP1) in the hemocytes of *H. roretzi*. The combination of expressed spots of HrSMLP1 distinguishes non-self cells from self cells with respect to CR inducibility. Given that the HrSMLP1 gene is a single gene, it may represent a novel type of self-marker protein with a role in CR.

## Background

Mechanisms for recognizing self and non-self cells play prominent roles in the immune system. Most metazoan cells possess an innate immune system that broadly classifies foreign bodies as non-self. In vertebrates, the major histocompatibility complex (MHC) is a widely recognized self-marker for allorecognition. Invertebrates lack lymphocytes and antibody-based humoral immune systems, and multicomponent adaptive immune systems may have evolved in primitive vertebrates [1, 2]. Lampreys and hagfish are primitive jawless vertebrates that are characterized by the initiation of specific immune responses [3]. Recently, a highly polymorphic membrane allogenic leukocyte antigen was identified as an MHC candidate in hagfish; its existence in a basal vertebrate suggests that all vertebrates possess MHC genes [4]. Immune responses in invertebrate are thought to represent precursors of the processes that contributed to the emergence of vertebrate immunity [5]. For example, several colonial marine invertebrates are capable of allorecognition [6–8], including sponges, which are among the most primitive metazoans [9]. Thus, even sponges are assumed to be able to recognize “histocompatibility” using self-markers. As forerunners of vertebrates, ascidians offer useful opportunities for studying the origins of allorecognition [10]. However, to date, no putative homologs of vertebrate MHC-encoding genes have been identified in the ascidian *Ciona intestinalis* [11, 12].

**Fig. 1:**
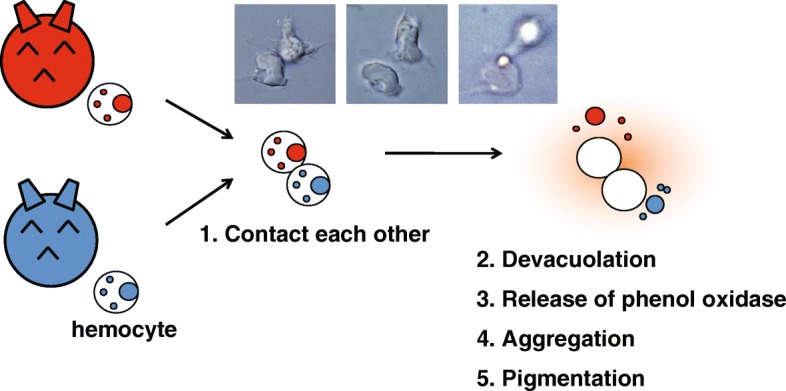
Scheme of the contact reaction in *Halocynthia roretzi.* When hemocytes from two cross-reactive individuals are mixed in vitro, they immediately release their vacuole contents and phenol oxidase, agglutinate, and become pigmented as part of the contact reaction (CR) process

Allorecognition in ascidians may represent a primitive form of vertebrate immunity [13]. Three types of allogeneic recognition systems are known in ascidians. In the first of these, colonial ascidians naturally undergo transplantation interactions (i.e., colony fusion) based on recognition of the origin of the interacting colonies [14–16]. This phenomenon is also known as histocompatibility, which has led to the proposal that these organisms may possess the ancestral molecular machinery for allorecognition. Such recognition occurs when two colonies of distinct origin meet in their habitat, or when samples from two such colonies are grafted under experimental conditions. In this regard, the *Botryllus* histocompatibility factor (*BHF*) locus in the ascidian *Botryllus schlosseri* has been identified as a polymorphic gene locus involved in colony fusion or rejection [17–19]. The second type of allorecognition is the avoidance of self-fertilization. Ascidians are hermaphrodites that spawn sperm and eggs simultaneously. Solitary ascidians, such as *C. intestinalis* [20] and *Halocynthia roretzi* [21], can prevent self-fertilization, and the latter is strictly self-sterile. Analysis of the genetic background of self-incompatibility in *H. roretzi* and *C. intestinalis* has yielded several intriguing insights [22–24]. The third type of allorecognition is “contact reaction” (CR) of hemocytes in *H. roretzi*. When hemocytes from different individuals are mixed in vitro, they immediately aggregate and extrude their vacuolar contents (Fig. 1) [25–27]. The CR process involves five steps. Initially, hemocytes from two individuals recognize and come into contact with each other (allogeneic recognition), several seconds after which de-vacuolation occurs. Thereafter, the hemocytes release phenol oxidase (PO) into the medium, which stimulates hemocyte aggregation at the reaction site and the formation of coagulates. Finally, the coagulating mass turns brown. This allogenic recognition pattern does not change through the *H. roretzi* life cycle.

It is possible to quantify the CR simply by measuring the activity of PO release [28]. Expression of the CR-inhibitory monoclonal antibody ku-4-96, which inhibits de-vacuolation, increases during the PO activity, coagulation, and pigmentation phases of the hemocytes of all individuals. This antibody is believed to exert its inhibitory action at an early stage in CR [29]. Hemocytes in *H. roretzi* have previously been classified into nine different cell types, and CR is primarily attributed to the action of vacuolated hemocytes, in which vacuoles occupy more than 50% of the cellular contents [30]. We hypothesized that self-marker molecules are present on the surface of hemocyte membranes, and that PO-inducing levels change in response to changes in the state of self-marker molecules. To elucidate the CR allorecognition mechanism, we produced monoclonal antibodies against insoluble membrane proteins isolated from the vacuolated hemocytes of *H. roretzi* and screened for monoclonal antibodies able to induce the release of PO from hemocytes derived from different individuals.

## Results

### Isolation of a PO release-inducing monoclonal antibody

**Fig. 2:**
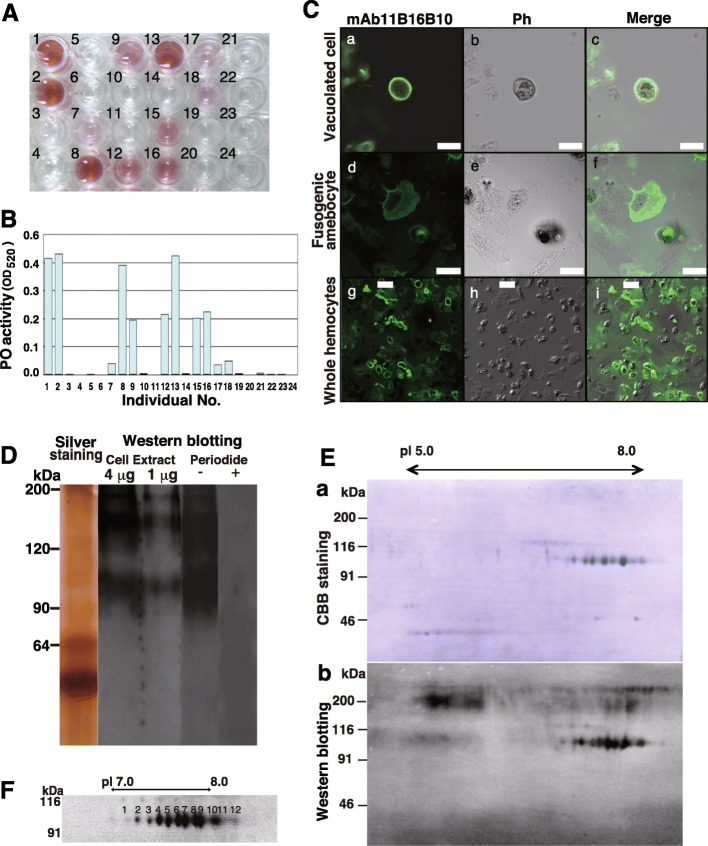
Immunochemical analysis performed with the phenol oxidase (PO)-releasing antibody mAb11B16B10. **A** mAb11B16B10-induced colorimetric PO activity of hemocytes from 24 individuals. **B** mAb11B16B10-induced OD_520_ values from **a** (*n* = 3). Numerals indicate individuals in **a**. **C** Immunostaining of hemocytes. Images of vacuolated cells **a**–**c**, fusogenic amebocyte **d**–**f** and whole hemocytes **g**–**i** are shown. mAb11B16B10 staining **a** and **d** and phase-contrast images **b** and **e** were combined to generate the merged images **c** and **f**. Scale bars in a–f = 10 μm. Scale bars in g–i = 20 μm. **D** Western blotting of hemocyte proteins using mAb11B16B10 (Cell Extract 4 μg and 1 μg) and the periodate treatment before (−) and after (+). **E** 2D-PAGE analysis of hemocyte proteins stained with Coomassie Brilliant Blue (CBB) **a** and immunoblotted with mAb11B16B10 **b**. **F** Enlarged typical view of the gel corresponding to a region encompassing 7.0–8.0 pI and 100 kDa

We prepared a PO release-inducing monoclonal antibody against an insoluble membrane protein of vacuolated cells. This yielded a hybridoma cell line that produced the IgGκ antibody mAb11B16B10. The PO assay results of the 24 individuals analyzed consistently revealed that the mAb11B16B10-induced PO activity differed among individuals (Fig. 2a, b).

### Characterization of an antigen for mAb11B16B10

We found that the antigen recognized by the monoclonal antibody mAb11B16B10 is a component of the *H. roretzi* hemocyte membrane. Immunostaining revealed that the mAb11B16B10 epitope is localized to the plasma membrane of vacuolated cells and the pseudopods of fusogenic amebocytes (Fig. 2c). Western blotting of a hemocyte plasma membrane fraction revealed that mAb11B16B10 bound to 100-, 170-, and 200-kDa proteins (Fig. 2d). Treatment of the plasma membrane fraction with periodate, which alters the structure of the sugar region by cleaving carbon–carbon bonds, abolished these western blot signals, indicating that mAb11B16B10 binds to sugar chains on all three protein variants. Furthermore, a combination of 2D-PAGE and western blotting revealed that mAb11B16B10 detected a series of 12,100-kDa protein spots that differed in pI (range, 7.0–8.0) among samples (Fig. 2e, f). However, no spots of approximately 170- and 200-kDa could be detected by 2D-PAGE using mAb11B16B10. These findings thus indicated that a group of 100-kDa proteins spots might contribute to the individual differences in PO activity.

### Identification of spots of 100-kDa proteins detected by mAb11B16B10

**Fig. 3:**
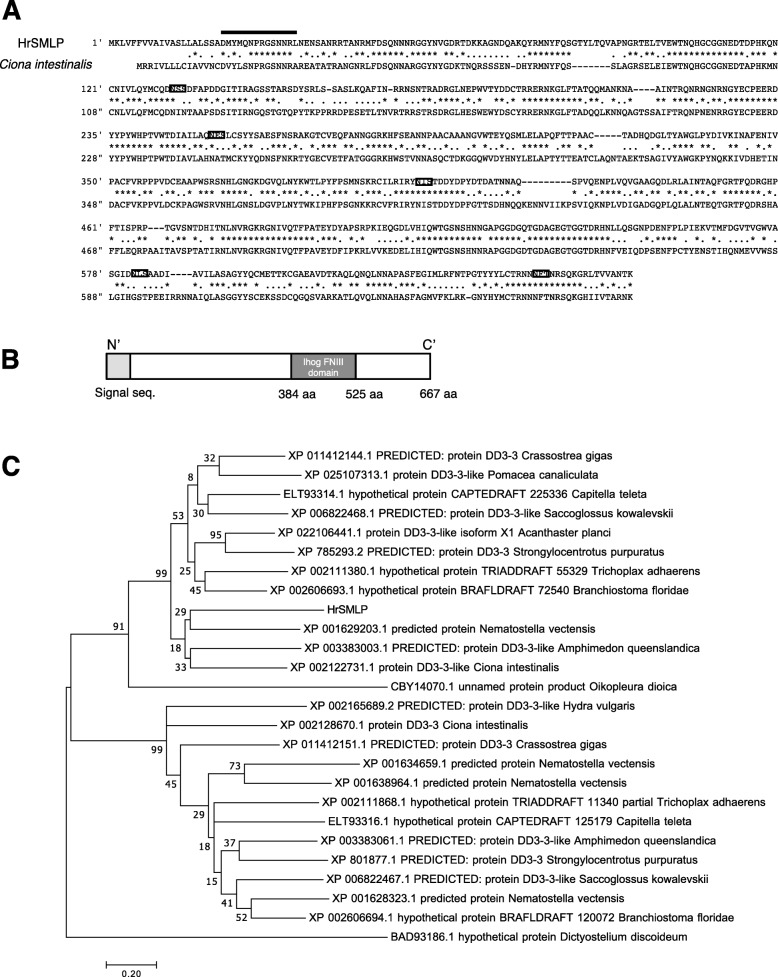
Sequence analysis of *100-kDa proteins detected by mAb11B16B10.* **a** 100-kDa proteins detected by mAb11B16B10 sequence compared with that of DD3–3-like protein from *C. intestinalis* (XP_002122731.1)*.* Bold line: Identical sequence determined using Edman degradation. Square box: N-glycosylation site. **b** Schematic of 100-kDa proteins detected by mAb11B16B10. **c** Phylogenetic analysis of HrSMLP1 and DD3–3-like proteins. The maximum-likelihood tree was constructed using MEGA version 7.0.26. DD3–3 protein from the social amoeba *Dictyostelium discoideum* represent the outgroup. The scale bar indicates the number of substitutions per site. Numbers at the nodes indicate the percentages of 1000 bootstrap replications (when higher than 50%). Accession numbers: *Crassostrea gigas* 1 (XP_011412144.1), *C. gigas* 2 (XP_011412151.1), *Pomacea canaliculata* (XP_025107313.1), *Capitella teleta* 1 (ELT93314.1), *C. teleta* 2 (ELT93316.1), *Saccoglossus kowalevskii* 1 (XP_006822468.1), *S. kowalevskii* 2 (XP_006822467.1), *Acanthaster planci* (XP_022106441.1), *Strongylocentrotus purpuratus* 1 (XP_785293.2), *S. purpuratus* 2 (XP_801877.1), *Trichoplax adhaerens* 1 (XP_002111380.1), *T. adhaerens* 2 (XP_002111868.1), *Branchiostoma floridae* 1 (XP_002606693.1), *B. floridae* 2 (XP_002606694.1), *Nematostella vectensis* 1 (XP_001629203.1), *N. vectensis* 2 (XP_001634659.1), *N. vectensis* 3 (XP_001638964.1), *N. vectensis* 4 (XP_001628323.1), *Amphimedon queenslandica* 1 (XP_003383003.1), *A. queenslandica* 2 (XP_003383061.1), *Ciona intestinalis*1 (XP_002122731.1), *C. intestinalis* 2 (XP_002128670.1), *Oikopleura dioica* (CBY14070.1), *Hydra vulgaris* (XP_002165689.2), *Dictyostelium discoideum* (BAD93186.1)

Edman degradation sequencing revealed that the partial N-terminal sequence of the 100-kDa proteins detected by mAb11B16B10 is DMYMQNPRGXNNRLNEN-. A BLAST query of the partial N-terminal amino acid sequences identified several genes, most notably XP_002122731.1 found in *C. intestinalis*. On the basis of the XP_002122731.1 sequence, we cloned the full-length cDNA of the 100-kDa protein (2344 bp, Fig. 3a). This sequence encoded a protein with a calculated molecular mass and pI of approximately 73.843 kDa and 6.44, respectively. Motif analysis revealed that the deduced polypeptide contains an N-terminal signal sequence and a domain structurally similar to the Ihog FNIII domain (Fig. 3b), suggesting that it may be a membrane protein. Moreover, the detection of five authenticated sites for *N*-glycosylation also indicates that it may be a glycoprotein. The sequence of this protein is highly similar to that of the DD3–3 protein from *Dictyostelium discoideum*. Phylogenetic analysis revealed that DD3–3-like proteins are present in invertebrates from Cnidaria to Chordata, but are not found in vertebrates (Fig. 3c). A search against the database of the Ascidian Network for in situ Expression and Embryological Data (ANISEED https://www.aniseed.cnrs.fr) revealed that this gene is located on scaffold 975 and comprises seven exons. 2D PAGE revealed that the 100-kDa protein band comprises 12 spots (pH 7.0–8.0, Fig. 2f). Notably, we detected a similarity in the partial N-terminal amino acid sequence of spot nos. 3, 4, 8, and 10 (DMXMQNP-; Additional file 1: Figure S1). Furthermore, genomic southern hybridization using this probe revealed a single band (Additional file 2: Figure S2), providing evidence that the cluster of spots detected by 2D-PAGE are encoded by the same gene. Moreover, these spots were found to be positive for Periodic acid–Schiff (PAS) staining, indicating that the proteins contain sugar moieties (Additional file 3: Figure S3).

### Differences in mAb11B16B10-induced PO activity among individuals

**Fig. 4:**
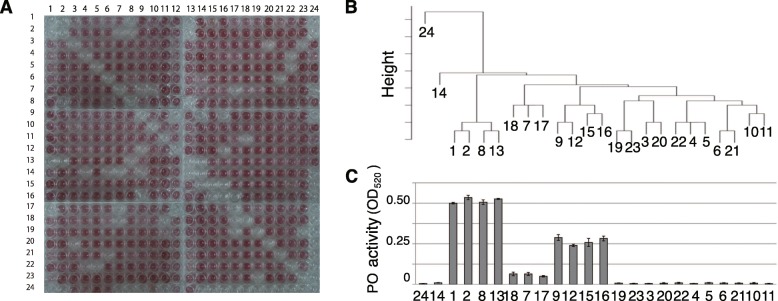
mAb11B16B10-induced PO activity and CR-induced PO activity in 24 individuals. **a** CR-induced colorimetric PO activity assays of hemocytes from 24 individuals. **b** Hierarchical clustering of the activities shown in **a** and Additional file 6: Table S1. **c** mAb11B16B10-induced OD_520_ values from Fig. 2B (*n* = 3)

An analysis of the CR-induced PO activity of 24 individuals (Fig. 4a and Additional file 6: Table S1) revealed the clustering pattern shown in Fig. 4b. mAb11B16B10 antibody-induced PO activity is shown in Fig. 2a, b. It appeared that the activities can be grouped into four clusters (a high-activity cluster: #1, 2, 8, and 13; a medium-activity cluster: #9, 12, 15, and 16; a low-activity cluster: #7, 17, and 18; and a no-activity cluster: 3, 4, 5, 6, 10, 11, 14, 19, 20, 21, 22, 23, and 24; *t* < 0.01; Fig. 4c). We also found that the level of PO activity induced by mAb11B16B10 corresponded closely to the division of these four clusters. These data accordingly indicate that the antigen recognized by mAb11B16B10 plays a self-recognition role in CR. We termed the antigen recognized mAb11B16B10 as HrSMLP1 (*Halocynthia roretzi* self-marker-like protein 1).

### HrSMLP1 sequence comparison among H. roretzi individuals

A comparison of the amino acid sequence of the HrSMLP1 from 24 individuals (Hr1–Hr24) (Additional file 4: Figure S4) revealed that sequences in 11 of these individuals (Hr1, Hr4, Hr6, Hr7, Hr9, Hr10, Hr12, Hr13, Hr17, Hr22, and Hr24) were identical, whereas a few unique substitutions were detected in the other individuals. To determine whether these substitutions play a role in CR, we compared the PO activity induced by CR in these individuals and their HrSMLP1 sequences. Individuals with the same amino acid sequence were distributed in four clusters by level of PO activity. The data revealed certain difference between the phylogenetic relationships and CR hierarchical clustering, indicating that the amino acid substitutions are unrelated to the allorecognition system.

### Detection of self-marker proteins through 2D-differential gel electrophoresis (2D-DIGE) using CR-trios

**Fig. 5:**
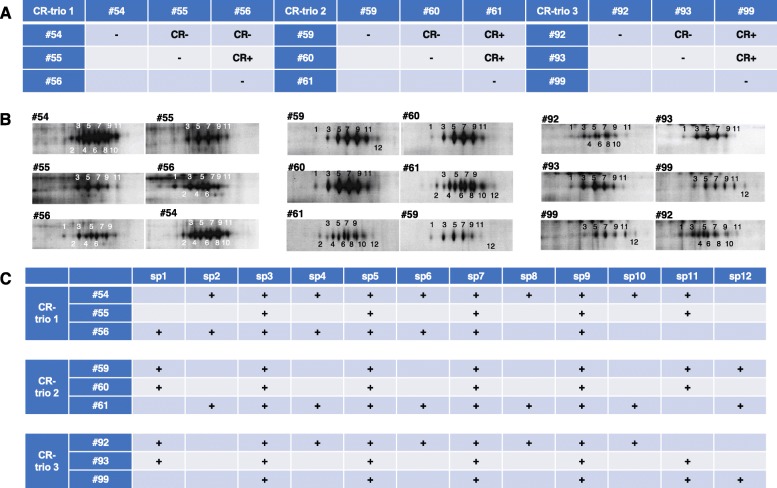
2D-differential gel electrophoresis (DIGE) of *H. roretzi* hemocytes using three CR-trios. (**a**) CR patterns between the different members of the *H. roretzi* CR-trios. (**b**) Gel section corresponding to the region encompassing 7.0–8.0 pI and 100 kDa of hemocyte proteins isolated from the individuals shown in **a**. Protein samples labeled with Cy3 (*left*) and Cy5 (*right*). **c** Summary of the 100-kDa protein isoforms (spots) found in each of the individuals (from **b**). Each of the 12 spots was classified by normalization with three 49 kDa protein spots (labeled with *yellow triangles* in Additional file 5: Figure S5)

It has previously been reported that CR occurs in 85% of contacts between distinct individuals of *H. roretzi* [25]. In the present study, we found that hemocytes of CR-non-reactive individuals harbor the same self-marker molecules, whereas the hemocytes of CR-reactive individuals are characterized by different self-marker molecules. On the basis of the results of the PO assays performed for 100 *H. roretzi* individuals, we selected three CR-trios (nine individuals in total) to distinguish CR-reactive/non-reactive individuals (Fig. 5a). We took advantage of the specific CR presentation patterns of three groups, each containing three individuals, referred to as “CR-trios 1–3,” which included CR-reactive and -non-reactive individuals, and subsequently performed 2D-DIGE to identify the series of twelve 100-kDa proteins (sp. 1–12). For each CR-trio, we searched for a series of protein spots shared among the CR-non-reactive individuals, but not among their reactive counterparts (Fig. 5b, c).

In CR-trio 1, consisting of individuals #54, #55, and #56, we found that CR did not occur between individuals #54 and #55 or between #54 and #56, but did occur between individuals #55 and #56 (Fig. 5a). In the 2D-DIGE analysis of the CR-non-reactive individuals #54 and #55, we observed that five spots (sp. 3, 5, 7, 9, and 11) were expressed in both individuals, whereas four spots were expressed in the CR-non-reactive individuals #54 and #56 (sp. 3, 5, 7, and 9) (Fig. 5b, c). In the comparative analysis of CR-reactive individuals #55 and #56, we found that eight spots (sp. 2, 3, 4, 5, 6, 7, 9, and 11) were expressed in only one of the two individuals.

In CR-trio 2, consisting of individuals #59, #60, and #61, CR did not occur between individuals #59 and #60, but it did occur between individual #61 and either #59 or #60 (Fig. 5a). 2D-DIGE analysis of the CR-non-reactive individuals #59 and #60 revealed that six spots (sp. 1, 3, 5, 7, 9, and 11) were expressed in both individuals, whereas comparative analysis between CR-reactive individuals #59 and #61 revealed that seven spots (sp. 1, 2, 4, 6, 8, 10, and 11) were expressed by only one individual. Similarly, for the comparison between CR-reactive individuals #60 and #61, we found that eight spots (sp. 1, 2, 4, 6, 8, 10, 11, and 12) were expressed by only one of the two individuals (Fig. 5b, c).

In CR-trio 3, consisting of individuals #92, #93, and #99, CR did not occur between individuals #92 and #93, but did occur between individual #99 and either #92 or #93 (Fig. 4a). In the comparative analysis of HrSMLP1 between individuals #92 and #93 (Fig. 4b, c), we detected five spots (sp. 1, 3, 5, 7, and 9) that were expressed in both individuals, whereas two spots (sp. 2 and 12) were not expressed in #92 and #93. In the comparative analysis between individuals #92 and #99, seven spots (sp. 1, 4, 6, 8, 10, 11, and 12) were found to be expressed by only one of the two individuals, and in the comparison between individuals #93 and #99, two spots (sp. 1 and 12) were expressed by only one of the two individuals.

All nine individuals assessed expressed sp. 3, 5, 7, and 9, and certain other spots (sp. 1, 2, 4, 6, 8, 10, 11 and 12) were expressed among the CR-reactive individuals.

## Discussion

**Fig. 6:**
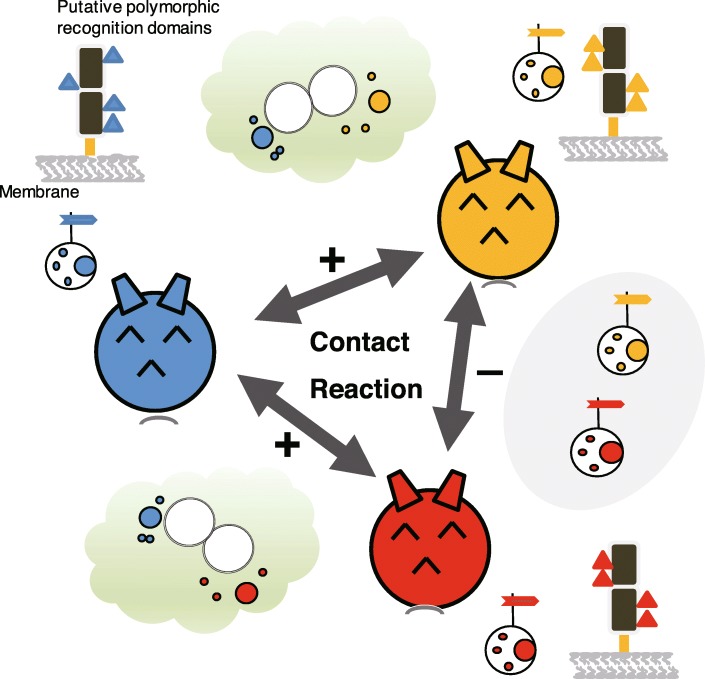
Overview of the process for identification of self-markers. CR is induced in 85% of contacts between individuals. Self-markers are likely membrane proteins with polymorphic recognition domains. The triangles showed polymorphic recognition domains. The patterns of the domains are different between CR induced individuals and similar between CR non-induced individuals

Presumably, any immune recognition molecule must both be expressed on the plasma membrane of cells and harbor polymorphic recognition domains. The molecular nature of self-markers should also be relatively similar, although not identical, across individuals within the same species (Fig. 6). The mechanism whereby natural killer (NK) cells recognize whether a cell is infected with a virus is explained by the missing “self” hypothesis [31, 32]. Previously, genomic analysis of urochordates failed to identify acquired immunity-related genes similar to MHC homologs [11, 12], suggesting that the self-markers involved in CR in *H. roretzi* are not MHC homologs. With regard to the hemocytes of *H. roretzi*, we found that CR does not occur between all individuals. The hemocytes of CR-non-reactive individuals display the same self-marker molecules, whereas the hemocytes of CR-reactive individuals display different self-marker molecules, which is consistent with the missing “self” hypothesis. Notably, patterns of HrSMLP1 variants differed among CR-reactive individuals, whereas they were virtually identical among their non-reactive counterparts. These results are consistent with our hypothesis that CR-non-reactive individuals express similar self-marker molecules on their plasma membrane, whereas CR-reactive individuals present self-marker molecules characterized by different modifications. Our comparison of the differences in membrane protein expression between CR-reactive individuals revealed that although different individuals displayed different spot variant patterns, all individuals maintained certain spot variants, namely, sp. 3, 5, 7, and 9 (Fig. 5). We subsequently examined the relationship between the spot variant patterns of individuals and the recognition of self or non-self by CR. With regard to CR-trio2, the CR-reactive pairs were #59 vs. #61 and #60 vs. #61. We identified differences in the patterns of sp. 1, 2, 4, 6, 8, 10, and 11 in these CR-reactive pairs, indicating that one of these seven spot variants was present in one individual of a pair and absent in the other individual. In contrast, all CR-non-reactive pairs showed the same variant pattern. With respect to CR-trio3, the CR-reactive pairs were #92 vs. #99 and #93 vs. #99, in which sp. 2, 4, 6, and 8 were common, but sp. 1 and 12 were different. In the CR-non-reactive pair #92 vs. 93, sp. 1 and 12 were common. With respect to CR-trio1, the CR-reactive pair was #55 vs. #56, in which sp. 1, 2, 4, 6, and 11 were different, whereas in the CR-non-reactive pairs (#54 vs. #55 and # 54 vs. #56), the variant group of sp. 1 and 11 or the variant group of sp. 2, 4, and 6 was common. These data thus provide evidence that two variant groups (sp. 1 and 11 and sp. 2, 4, and 6) may play an important role in allorecognition. As sp. 1 was detected in one member of each of the CR-reactive pairs, we suspect that it may be an effective variant for CR induction. These results are consistent with our hypothesis that CR-non-reactive individuals express similar self-marker molecules on their plasma membrane, whereas CR-reactive individuals present self-marker molecules with different modifications.

We observed that the monoclonal antibody mAb11B16B10 stimulated PO release to differing extents in the ascidians and identified a series of 100-kDa protein variants with distinct pIs (Fig. 2e). Hierarchical clustering revealed that the levels of CR-induced PO release observed in the 24 individuals studied can be grouped into four clusters (Fig. 4b, c), by the respective PO activity levels observed following mAb11B16B10 stimulation. These data support our assumption that the 100-kDa proteins are involved in the CR of *H. roretzi* hemocytes. The 100-kDa protein HrSMLP1 identified in the present study shows similarities with the DD3–3 protein of *Dictyostelium discoideum* and DD3–3-like proteins present in other invertebrates, but not in vertebrates. DD3–3 contain five *N*-glycosylation and two phosphorylation sites (Fig. 3) and is probably involved in some aspect of glycosylation recognition [33, 34]. Furthermore, exhaustive transcriptional analyses focused on immune-related molecules has led to the identification of a DD3–3 homolog in sponges [35]. Notably, mAb11B16B10 binds to sugar chains on HrSMLP1 (Fig. 2d) and HrSMLP1 contains *N*-glycosylation site similar to those characterizing DD3–3.

The glycan linkages of mammalian hosts are abundant and attractive targets for pathogens to establish infections via lectin binding. A subset of Toll-like receptors and C-type lectins function to detect the glycans of lower organisms as a means of non-self discrimination and immunological activation [36]. In addition, the C-type lectin superfamily member CD94, or the CD94/NKG2A heterodimer, harbors carbohydrate recognition domains in the extracellular region and recognizes MHC class I molecules on potential target cells. Furthermore, CD94/NKR-P1-related molecules have previously been identified in *Botryllus* hemocytes [37]. Consistent with these findings, our data show that HrSMLP1 contains sugar moieties that serve as epitopes for mAb11B16B10 (Fig. 6 and Additional file 3:Figure S3). We found that all 12 variants of the HrSMLP1 protein are glycoproteins derived from the same gene, and we speculate that they may differ with respect to changes in the glycan linkages at the individual level, indicating that HrSMLP1-mediated CR resembles the NK cell response.

Self-fertility in ascidians has been described previously, and in *H. roretzi* and *H. aurantium*, the HrVC70 and HaVC80 proteins have been shown to determine self-sterility [21, 22]. Similarly, three highly polymorphic loci, *Themis-A*, *Themis-B*, and *vCRL1*, have been reported in *C. intestinalis* [23, 24, 38, 39], although allorecognition has not been observed in *C. intestinalis* hemocytes. Although the expression of *Themis-A*, *Themis-B*, and *vCRL1* is mediated by genomic polymorphisms, our data indicate that HrSMLP1 is regulated by post-translational modifications (Additional file 3: Figure S3). Thus, HrSMLP1 may represent a novel type of self-marker molecule involved in invertebrate allogeneic recognition. However, the regulation of post-translational modifications cannot be controlled at the genome level, and these modifications are not inherited across generations. Therefore, the evolution of self-markers in vertebrates may not be based on a system like that of HrSMLP1. It appears that the evolution of molecules that undergo various self and non-self recognition progressed until the evolution of the acquired immune system of vertebrates. Evidently, HrSMLP1 may be a product of several alternative outcomes that occurred over the course of evolution.

## Conclusions

The monoclonal antibody mAb11B16B10 can induce the release of PO from hemocytes derived from different individuals; however, the induced PO activity differs among individuals. This antibody recognizes a group of 100-kDa membrane proteins in the hemocytes of *H. roretzi*, which have high homology with the DD3–3 protein, which is an immune-related molecule in the sponges. Combinations of detected 100-kDa protein spots differed among self and non-self cells distinguished by CR inducibility. These proteins were collectively named HrSMLP1, which may be a novel type of self-marker protein involved in CR.

## Methods

### Animals and hemocyte collection

Solitary ascidians *H. roretzi* (type C) were collected from the coast of northern Honshu Island, Japan, at Mutsu Bay, Aomori Prefecture. Hemocytes were collected as described previously [28].

### MHI assay and PO-activity measurement

CR and PO activities were measured using mixed-hemocyte incubation (MHI;) and colorimetric assays respectively, as previously described, with slight modifications [28, 37]. CR was induced in the U-bottom wells of 96-well plates by separately mixing 100 μL of the hemocyte suspension (4–8 × 10^6^ cells/mL) obtained from each ascidian in artificial seawater (ASW) or Mg^2+^-free ASW containing 506 mM NaCl, 10.6 mM KCl, 11.5 mM CaCl_2_, 6.07 mM NaHCO_3_, pH 8.0. After incubation for 2 h at room temperature (RT), the plates were centrifuged in a swing rotor (100×*g*, 10 min), and the supernatants were assayed at RT for PO activity.

### H. roretzi hemocyte membrane collection

Hemocytes were collected from the dissected tunic matrix of the ascidians, without injuring the internal organs, and transferred into 50 mL of marine anticoagulant (13 mM citric acid, 10 mM EDTA, 0.45 M NaCl, 0.1 M glucose, 15 mM trisodium citrate, pH 7.0). After centrifugation in a swinging bucket rotor (100×*g*, 4 °C, 18 min), hemocytes were collected, and the process was repeated three times. Hemocytes were homogenized using a glass pestle in 1/2× phosphate-buffered saline (PBS) containing a protease-inhibitor cocktail (Complete; Roche Applied Science, Indianapolis, IN). The homogenate was centrifuged (20,000×*g*, 30 min), the supernatant containing the soluble material was discarded, and the pellet was suspended in 1/2× PBS. This step was repeated twice to thoroughly wash the pellet, which was then dissolved in lysis buffer (8 M urea, 2 M thiourea, 4% CHAPS, 40 mM Tris base, plus the protease-inhibitor cocktail), and homogenized using a glass pestle and disrupted using a sonicator (ULTRASONICS100–132–134; Branson, Emerson, St. Louis, MO). After ultracentrifugation (250,000×*g*, 20 min; Optima TLX, Beckman), the supernatant, which contains hemocyte membranes, was collected.

### Monoclonal antibody production

Vacuolated cells were obtained using discontinuous bovine serum albumin (BSA) density-gradient centrifugation [32]. Insoluble membrane proteins isolated from the vacuolated cells were used to immunize a C3H/HeNCrj mouse (age: six weeks). Monoclonal antibodies were produced using a hybridoma technique as previously described [33].

### Immunocytochemistry

Hemocytes were allowed to adhere (90 min, RT) to micro cover glasses (Matsunami, Osaka, Japan) in 6-well plates (BM Equipment, Tokyo, Japan). After removing the ASW, hemocytes were blocked (30 min, RT) with Ca^2+^/Mg^2+^-free ASW (CMFASW) containing 1% BSA; the composition of CMFASW was 480 mM NaCl, 9.4 mM KCl, 32 mM Na_2_SO_4_, 3.2 mM NaHCO_3_, 10 mM EPPS, pH 8.0. The primary antibody (mAb11B16B10) and secondary antibody (Alexa Fluor 488 rabbit anti-mouse IgG; Molecular Probes, Thermo Fisher Scientific, Waltham, MA) were diluted to 1 and 0.4 μg/mL, respectively, in 2 mL of CMFASW containing 1% BSA, and added sequentially to each well and incubated (30 min, RT); the hemocytes were washed five times with CMFASW containing 1% BSA between incubations, and three times with ASW after incubation with the secondary antibody, and then examined under a fluorescence microscope (AXIO Imager M1; Zeiss, Oberkochen, Germany).

### Electrophoresis and western blotting

SDS-PAGE and native PAGE were performed on 7.5% gels, and proteins were stained with CBB. Glycoproteins were stained by peroxidase oxidation and Periodic acid–Schiff stain (PAS staining). For western blotting, proteins were transferred onto PVDF membranes (Hybond-P; GE Healthcare), and the membranes blocked with TBS-T (Tris-buffered saline plus 0.1% Tween 20) containing 5% skimmed milk, and then incubated with 0.2 μg/mL mouse mAb11B16B10 (4 °C, overnight). Next, the membranes were washed with TBS-T, incubated with 0.04 μg/mL horseradish peroxidase-conjugated goat anti-mouse IgG antibody (30 min, RT), and then washed again. The bound antibody was detected using ECL Plus (GE Healthcare) and a Molecular Imager FX (Bio-Rad).

### 2D-PAGE and western blotting of H. roretzi hemocyte membrane proteins

Excess salts and other impurities were removed from each sample set using a Ready Prep 2D Clean Up kit (Bio-Rad, Hercules, CA, USA) as per the manufacturer’s protocol, and protein concentrations were measured using a Quick Start Bradford Dye Reagent Protein Assay kit (Bio-Rad). For 2D-PAGE (2D polyacrylamide gel electrophoresis), 50 μg of protein samples were used to rehydrate 11-cm, pH 5–8 Ready Strip™ IPG strips (Bio-Rad) overnight in rehydration buffer: 8 M urea, 2 M thiourea, 2% CHAPS, 1.5 M Tris-HCl (pH 8.8), BPB, 0.35% dithiothreitol (DTT), 0.5% IPG buffer (pH 3–10; GE Healthcare, Pittsburgh, PA, USA). Rehydrated strips were subjected to IEF by using an Electrophoresis Power Supply EPS 3501 XL (GE Healthcare) at 20 °C throughout focusing, and then at 250 V for 15 min, 3500 V for 2 h 50 min, 3500 V for 12 h 57 min, and 500 V for 15 min, after which the strips were equilibrated (30 min) in equilibration buffer (50 mM Tris-HCl (pH 8.8), 6 M urea, 30% (v/v) glycerol, 2% (w/v) SDS, with BPB). SDS-PAGE was conducted with 7.5% gels, and proteins were stained with Coomassie Brilliant Blue (CBB) or by silver staining.

### N-terminal amino acid sequencing

Self-marker protein candidates were subjected to isoelectric focusing (IEF; pH 3–10) for greater resolution, and isolated for sequencing.

After SDS-PAGE, proteins were transferred onto polyvinylidene difluoride (PVDF) membranes (GE Healthcare), and the blotted proteins were excised from the membrane and sequenced through automated Edman degradation using a Procise 492 (PerkinElmer, Waltham, MA, USA) protein sequencing system at the Instrumental Analysis Division, Creative Research Institution of Hokkaido University.

### Full-length cDNA cloning

To obtain full-length cDNAs, we designed degenerate PCR primers based on the XP_002122731.1 sequence (*C. intestinalis*). Total RNA was extracted and purified, and then cDNAs were synthesized as described [29] and amplified by performing RT-PCR (listed in Support information) and RACE-PCR with a SMART RACE cDNA Amplification Kit (BD Biosciences Clontech, Franklin Lakes, NJ). Nucleotide sequences were determined using the dideoxy method with a BigDye Terminator Cycle Sequencing Ready Reaction Kit (Applied Biosystems, Waltham, MA) and an ABI PRISM 3100 Genetic Analyzer (Applied Biosystems). All new sequences were subjected to similarity searches on the National Center for Biotechnology Information BLAST databases, and multiple sequences were aligned using ClusterW (https://www.genome.jp/tools-bin/clustalw). Information on conserved protein domains was found using InterProScan (http://www.ebi.ac.uk/Tools/InterProScan/) and TMHMM (http://www.cbs.dtu.dk/services/TMHMM/).

### Construction of a phylogenetic tree

Phylogenetic analysis was performed in MEGA version 7.0.26. The amino acid sequences used for the analysis are listed in Additional file 6: Table S1. A multiple-sequence alignment was first performed using the MUSCLE algorithm with the default parameters. Subsequently, a maximum likelihood tree was constructed with 1000 bootstrap replicates. The best model LG + G + I was selected using Akaike information criterion. Gaps were eliminated and thus not included for the analysis.

### Clustering analysis

Static analysis was performed using the R software environment. CR-induction and CR-non-induction were defined as 1 and 0, respectively. Each individual’s relationships were expressed as a square matrix, the Manhattan distance was calculated, and clustering was predicted using the group average method (*n* = 3). The threshold for CR induction was set at 0.13.

### 2D-differential gel electrophoresis (2D-DIGE) of hemocyte membrane proteins

Supernatant samples were labeled with the cyanine dyes, Cy3 or Cy5, as per the manufacturer’s guidelines (GE Healthcare), with minor modifications. Before IEF, 50-μg aliquots from each of the three labeling mixtures were combined, and then mixed with equal amounts of 2× sample buffer (7 M urea, 2 M thiourea, 4% CHAPS, 2% Pharmalyte (pH 3–10; GE Healthcare), 2% DTT). Next, rehydration buffer (7 M urea, 2 M thiourea, 4% CHAPS, 1% Pharmalyte, 0.2% DTT) was added to obtain a final volume of 450 μL, and then each sample was used for in-gel rehydration of Immobiline DryStrip 24-cm strips (pH 3–10; GE Healthcare) in rehydration buffer (12 h, RT), as per the manufacturer’s instructions. IEF was performed using an Ettan IPGphor II (GE Healthcare) at 500 V for 1 h, 1 kV for 1 h, and 8 kV for 7.5 h. Before electrophoresis, each immobilized-pH-gradient strip was reduced (for 15 min each) in buffer A (100 mM Tris, 6 M urea, 30% glycerol, 2% SDS, 0.5% DTT), followed by buffer B (100 mM Tris, 6 M urea, 30% glycerol, 2% SDS, 4.5% iodoacetamide). Second-dimension 7.5% SDS-PAGE was run on an Ettan DALTsix Large Electrophoresis System (GE Healthcare) for 4 h at 600 V, 400 mA, 4 W/gel. Analytical gels were stained overnight with SYPRO Ruby (Molecular Probes, Thermo Fisher Scientific, Waltham, MA, USA) according to the manufacturer’s instructions. All images were collected on a Typhoon 9400 (GE Healthcare). Statistical analysis and quantification of protein expression were performed using DecyderDIA software (GE Healthcare).

## Supplementary information

**Additional file 1: Figure S1.** N′ terminal sequence of each spot of self-marker candidate proteins. Spot no. 3, 4, 8 and 10 were excised from the membrane and sequenced by automated Edman degradation at the Instrumental Analysis Division, Creative Research Institution of Hokkaido University.

**Additional file 2: Figure S2.** Genomic southern hybridization of 100-kDa proteins crossed by mAb11B16B10. *H. roretzi* genome DNAs were digested with *Bam*HI or *Pst*I and performed southern hybridization with HrSMLP1 probe.

**Additional file 3: Figure S3. **PAS staining of self-marker candidate proteins. The membrane extract of *H. roretzi* hemocyte were performed 2D-PAGE stained by CBB staining (left panel) and stained by peroxidase oxidation and Periodic acid–Schiff stain (right panel).

**Additional file 4: Figure S4.** Polymorphism analysis in HrSMLP1. HrSMLP1 transcripts in 24 individuals (Hr.1–24) were sequenced and compared. The red letters show the substitution sites and amino acids in comparison with Hr.1 Accession numbers: Hr.1 (LC488798), Hr.2 (LC488799), Hr.3 (LC488800), Hr.4 (LC488801), Hr.5 (LC488802), Hr.6 (LC488803), Hr.7 (LC488804), Hr.8 (LC488805), Hr.9 (LC488806), Hr.10 (LC488807), Hr.11 (LC488808), Hr.12 (LC488809), Hr.13 (LC488810), Hr.14 (LC488811), Hr15 (LC488812), Hr.16 (LC488813), Hr.17 (LC488814), Hr.18 (LC488815), Hr.19 (LC488816), Hr.20 (LC488817), Hr.21 (LC488818), Hr.22 (LC488819), Hr.23 (LC488820), Hr.24 (LC488821)

**Additional file 5: Figure S5. **2D-diffrerential gel electrophoresis (DIGE) of *H. roretzi* hemocytes membrane proteins. Left panel: individuals #61, right panel: individuals #59

**Additional file 6: Table S1.** Raw data of PO activity on CR between Hr.1–24

## Data Availability

Please contact author for data requests.
